# Experiences of a multistep process with medical and psychological interventions for patients with congenital uterine aplasia to achieve motherhood: the Gothenburg–Tübingen collaboration

**Published:** 2019-06

**Authors:** SY Brucker, F-A Taran, K Rall, D Schöller, P Dahm-Kâhler, N Kvarnström, S Järvholm, S Nadalin, A Königsrainer, D Wallwiener, M Brännström

**Affiliations:** Department of Obstetrics and Gynecology, University of Tübingen, Tübingen, Germany; Department of Obstetrics and Gynecology, Sahlgrenska Academy, University of Gothenburg, Göteborg, Sweden; Stockholm IVF, Stockholm, Sweden; Department of Transplantation, Sahlgrenska Academy, University of Gothenburg, Göteborg, Sweden; Department of General, Visceral and Transplant Surgery, University of Tübingen, Tübingen, Germany

**Keywords:** congenital uterine and vaginal aplasia, laparoscopic-assisted neovagina, MRKH-Syndrome, Vecchietti, uterus transplantation

## Abstract

Congenital uterine aplasia, also known as Mayer–Rokitansky–Küster–Hauser syndrome (MRKHS) is a condition associated to a non-functional uterus in the presence of functional ovaries. In a setting where surrogacy is illegal (or not accepted) and adoption is the only alternative, neovaginoplasty and subsequent uterus transplantation (UTx) can provide a route to motherhood for women with MRKHS. This review article describes a multistep process by which patients with MRKHS can achieve motherhood with their own biological child. This process involving a careful clinical diagnosis, psychological counselling, assessment of eligibility for neovagina creation and UTx, the surgical treatment, fertility treatment, and long-term follow-up was developed at the Tübingen University Hospital and in close collaboration with Sahlgrenska Academy, University of Gothenburg, Sweden, where the basic experimental and clinical groundwork for UTx was laid and the first-ever UTx procedure was performed.

## Introduction

The primary aim of this review is to describe a multistep process enabling patients with congenital uterine aplasia to achieve motherhood and deliver their own biological child in a setting where surrogacy is illegal and adoption is the only alternative. Also, we address the required team set-up and report the initial clinical experience of a first uterus transplantation (UTx) program established in Germany.

## The goal of being able to have vaginal intercourse

The lack of centralization of patients with rare diseases is omnipresent in almost every healthcare system. Hence, misdiagnosis or symptomatic treatment without having an identified underlying disease process can occur. Health care providers in general practice and subspecialists are equipped to diagnose commonly seen diseases. As such, most practitioners have little or no experience with rare diseases. Girls with Mayer–Rokitansky–Küster–Hauser syndrome (MRKHS) develop normal secondary sexual characteristics, including breast growth, body proportions, body hair, and hymenal tissue. Thus, a primary diagnosis is generally made relatively late, often at the onset of puberty when menstruation fails to occur or, less frequently, when a patient is unable to have vaginal sexual intercourse (Wagner et al., 2016). As a result, these young women experience a lasting negative impact on their self-esteem and self-image during the sensitive phase of puberty (Heller-Boersma et al., 2009; Wagner et al., 2016). After diagnosis, patients should be referred to a specialized center that offers both medical and psychological support. This involves the creation of a functional neovagina and subsequent counselling about their sexual life and infertility. The Tübingen Center for Rare Female Genital Malformations was inaugurated as part of the first German Center for Research and Treatment of Rare Diseases in 2010, with the intention of providing comprehensive and holistic support for patients from diagnosis to therapy.

The creation of a functional neovagina that enables the patient to have vaginal intercourse, providing adequate cosmetic results while minimizing short and long-term morbidity. This procedure is currently considered the primary therapeutic goal in patients with MRKHS (Brucker et al., 2008; Rall et al., 2014). Our group developed and optimized a minimally invasive technique that creates a neovagina in a standardized, controlled manner by vaginoabdominal blunt perforation and subsequent traction exerted by a device positioned on the patient’s abdomen (Brucker et al., 2008; Rall et al., 2014). The procedure is fast, effective, and minimally traumatic, and has a very low complication rate with excellent long-term functional results, including the correct anatomical axis (Rall et al., 2014). An alternative to surgical neovagina creation known as Frank’s method, which involves self-dilation using a vaginal dummy, is available but considered tedious and painful, plus, requires discipline, self-motivation, persistence, and determination on the part of the patient (Nakhal & Creighton, 2012).

## The goal of becoming a mother

Surgical or non-surgical creation of a neovagina alone does not ensure a successful psychological outcome in women with a diagnosis of MRKHS (Bean et al., 2009). Several studies reported infertility as one of the least acceptable aspects of the condition, since childlessness can be psychologically stressful for the affected women (Bean et al., 2009). Moreover, the further well-being over time of women with MRKHS after neovagina creation is greatly influenced by the additional aspect of infertility (Wagner et al., 2016). It is therefore of great importance to discuss the available options with the patients and their families.

Until recently, for these women the only possibility to achieve motherhood were adoption or surrogacy, i.e. the use of a gestational carrier. However, the latter option is currently illegal in Germany and the Nordic countries, and in many other countries and societies worldwide (Deutsch, 1992; Brännström, 2015). Due to the legislation in these countries, UTx is the only future option these women have to conceive their own biological child. Therefore, in our opinion, UTx is a viable alternative for surrogacy, which would include cross-border reproduction between our countries. Our personal experience has evidenced that many patients with MRKHS opt for UTx because they wish to be able to become pregnant and carry their own child them to become emotionally attached to their child right from the beginning of pregnancy, strong arguments in favor of UTx. Plus, to experience pregnancy is also seen as an important part of a patients’ self-image.

UTx can be performed with an organ obtained from either a live or a deceased donor. The advantages of live-donor UTx, as performed in the Tübingen UTx program, is that the quality of the transplanted organ may be superior, mainly because the organ can be meticulously assessed preoperatively and an optimal timing of surgery can be achieved. The latter is important in order to assemble the designated team and to ensure the shortest ischemic time possible.

However, all live donor situations involve the additional risks associated with donor surgery, which raise ethical concerns. While there are no reports of persistent physical impairment or life-threatening events in uterus donors, serious complications including a reoperation have been described (Brännström, 2015; Testa et al., 2016; Kvarnström et al., 2017). In addition, there seems to be a psychological risk for the donor regarding a negative outcome for the recipient, including the event of a prolonged period without the birth of a child (Kvarnström et al., 2017; Järvholm et al., 2015).

## Preliminary set-up for the first UTx in Germany

**Figure 1:**
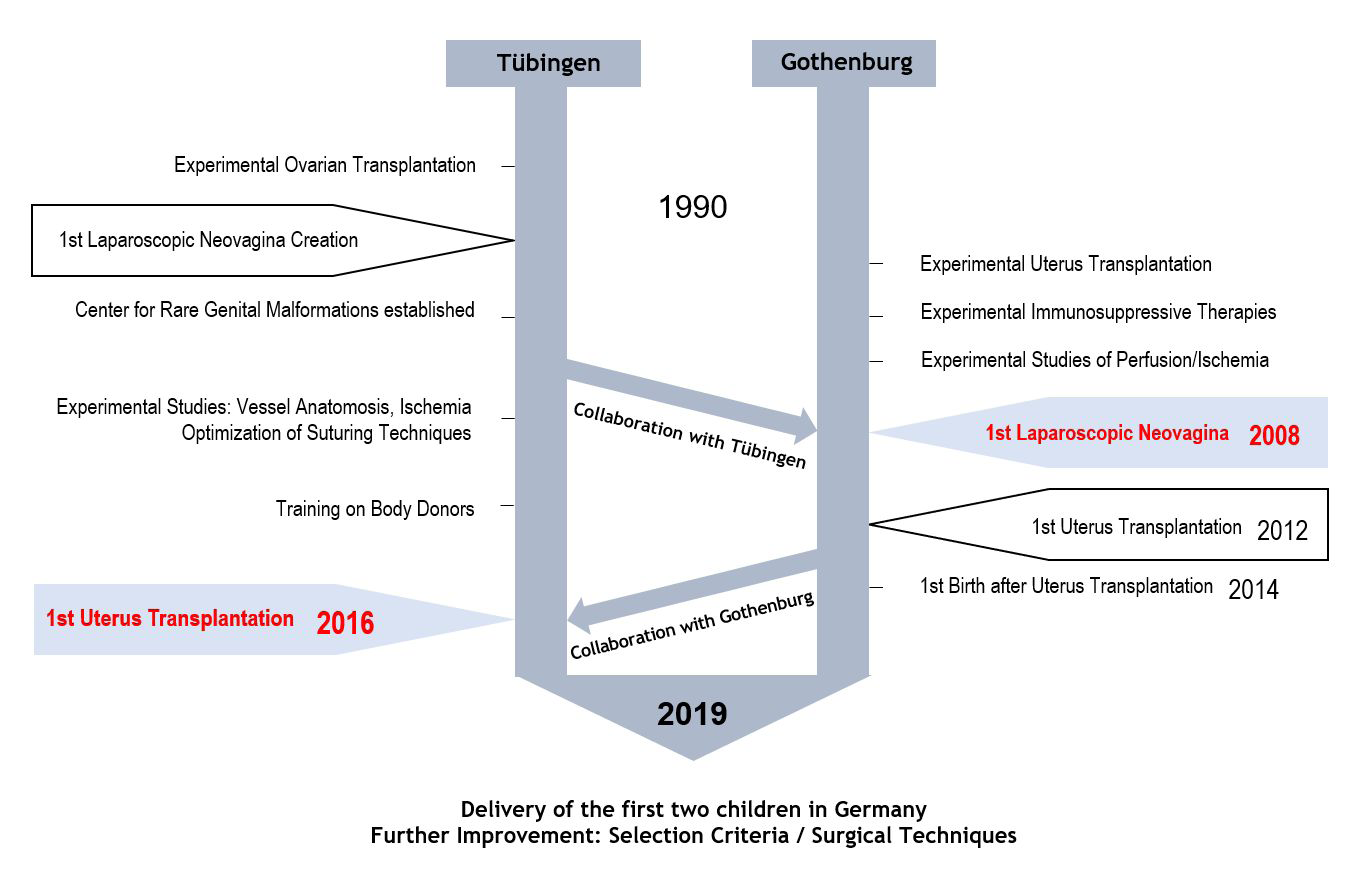
— The Gothenburg–Tübingen Collaboration: Neovagina Creation / Uterus Transplantation

Human UTx was introduced in Sweden after meticulous and systematic animal-based research for more than a decade in rodents, domestic species, and non-human primate species (Brännström et al., 2012; Akhi et al., 2013; Diaz-Garcia et al., 2013; Johanesson et al., 2013; Diaz-Garcia et al., 2014; Tryphonopoulos et al., 2014). This is most likely an essential factor for the positive results of the initial observational study of human UTx where six out of nine included women achieved motherhood (Mölne et al., 2016). Five of these six women, had one or two live births after UTx, had MRKHS and one had no uterus due to radical hysterectomy for early stage cervical cancer. The entire transformation process from being a girl with MRKH to becoming a woman capable of having vaginal intercourse and experiencing pregnancy and childbirth by undergoing neovaginoplasty and subsequent UTx, and the vision of woman with absolute uterine factor infertility (AUFI) becoming a mother by UTx, was preceded by several years of basic experimental and clinical research through a close collaboration between the Swedish and German multidisciplinary groups in Göteborg (Gothenburg) and Tübingen. The main focus of the Tübingen group, who had conducted initial research in ovarian transplantation and microsurgery techniques, was to optimize the neovagina creation procedure and to implement and widely disseminate their minimally invasive neovaginoplasty method (Wallwiener et al., 1990; Wallwiener et al., 1991; Scheidel et al., 1982; Scheidel et al., 1986; Rimbach et al., 1993; Gauwerky et al., 1992; Brucker et al., 2008; Rall et al., 2014). The Göteborg group initially focused on animal-based and later on pre-clinical UTx research, which was ultimately followed by the first clinical trial of UTx, launched 2012. The collaboration between our two working groups began a decade ago when the first laparoscopic neovagina procedure was performed in Göteborg and reached its pinnacle when the first-ever UTx in Germany was carried out in October 2016 (Figure 1).

Planning of the initial setting (preliminary experimental and clinical research activities and development of the team) and profound analysis of the psychosocial and ethical background for UTx has been ongoing for several years at the Departments of Women’s Health and Transplantation Surgery in Tübingen. This was conducted in close collaboration with the Göteborg team, who were the pioneers and experts in UTx but less experienced in respect of the specific psychological and quality-of-life issues faced by women with MRKHS. Consequently, the first clinical UTx program in Germany was initiated at the Tübingen University Hospital in 2016. The process incorporated advice from several institutions: the Ethical Committee of the University of Tübingen, the Standing Organ Transplantation Committee of the German Medical Association, the Baden-Württemberg State Board of Physicians, the Living Donor Committee of the District Chamber of Physicians, and the Multidisciplinary Transplantation Board of Tübingen University Hospital. The UTx program was launched on January 23rd, 2016 during the sixth Symposium dedicated to patients with AUFI held at Tübingen University Hospital. The symposium was attended by a total of 166 participants, the majority being women with MRKHS, but also their partners and parents as well as health-professionals who had a professional interest and role in the management of women with AUFI, particularly those with MRKHS.

## Development of the multidisciplinary team

**Figure 2:**
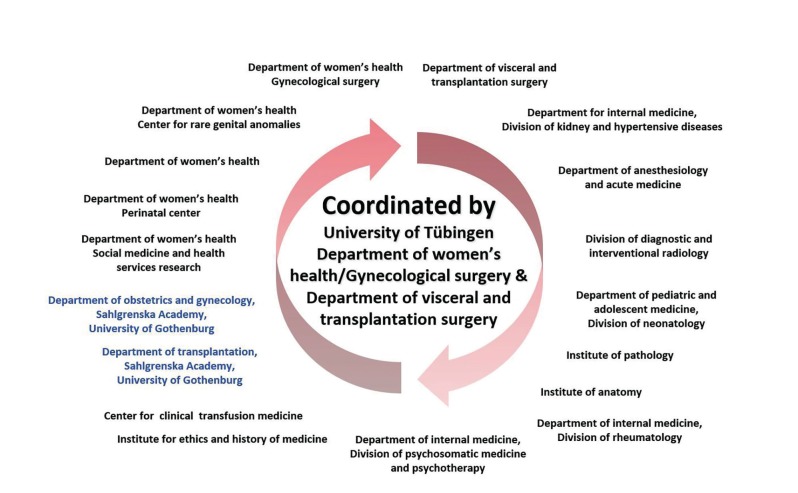
— The Gothenburg–Tübingen multidisciplinary uterus transplantation team .

In the case of UTx, the team and center must not only have the infrastructure to deal with the longitudinal aspects of the surgical procedure and its consequences, which may span several years until the delivery of a baby, but should also master the surgical techniques involved, the complex preoperative investigations of future uterus recipients, their partners, and the donors (Flyckt et al., 2016). Additionally, the functional outcome of a transplanted uterus includes the monitoring of multiple parameters, which must take place before pregnancy can occur and proceed to a healthy newborn (Flyckt et al., 2016). Thus, similar to other UTx programs, our team includes, amongst others, members from the following specialties: gynecologic surgery, transplantation surgery, reproductive medicine, maternal-fetal medicine, neonatology, internal medicine, psychiatry and psychotherapy, anesthesiology, radiology, and pathology (Figure 2). However, the UTx program in Tübingen also places particular emphasis on the psychological aspects; e.g. specific questionnaires and interviews were used according to the Swedish study, accompanied by long-term psychological follow-up before and after the transplantation or exclusion from the program for medical reasons. The ethical background to, and implications of, the procedure were taken into consideration by involving the director of the University of Tübingen’s Institute for Ethics in Medicine, placing the focus on German transplantation law and its application to UTx. As a result, the final team comprised a total of 40 physicians from 18 university departments, institutes, and centers (Figure 2).

As previously described, a psychological pre-transplantation assessment was performed to determine the participants’ suitability and excluding individuals found to be under severe psychological strain (Järvholm et al., 2015). At this early phase of medical trials, candidates are in a way, self-selecting in favor of stability. This was also the case in the first Swedish cohort of recipients and partners, who, regarding to psychosocial well-being at inclusion, were all found to be comparable to the general population, or better. Thus, all patients underwent extensive psychological and psychosocial counselling. Also, the involvement of the Tübingen Institute of Medical Ethics from the early prearrangements for the program was considered of fundamental importance (Figure 1). In one case, the pre-UTx interviews revealed that the donor was no longer sure of her wish to undergo the procedure and thus was excluded with given medical reasons as an excuse, in accordance with the procedure chosen in other cases, e.g. living kidney donation (unpublished data). All remaining 13 included donor-recipient pairs were psychologically stable and well-enough adjusted to take part in the UTx program (Taran et al., 2019).

## Initial clinical experience of human live donor transplant

Recently, we reported the details of all cases discussing modifications to the selection and inclusion criteria for our program in a separate publication (Taran et al., 2019). The latter case, however, underscores the importance of a robust, well-prepared setting for UTx. Furthermore, as highlighted by Flyckt et al. (2016), well-selected patients with strong social and clinical support will be better equipped to handle the psychosocial aspects of transplant success and failure. The experiences gained from the Swedish study concerning the psychological aspects of post-UTx procedures for recipients and their partners over a 1-year period after UTx have been reported by Järvholm et al. (2015). Their main findings were that, although there were slight deviations in psychosocial well-being post surgery, all data returned to baseline during the first year. This was also the case for the two couples who experienced graft loss.

Medical and psychological results of a one-year follow-up of live donors in the original Swedish study have been published by Kvarnström et al. (2017). Their major findings were that donors returned to the baseline values of their Health Related Quality of Life and to normal levels of anxiety and depression (Hospital Anxiety and Depression Scale (HADS) score) even though negative events had occurred, such as one case of ureter reimplantation and two cases of transient local symptoms.

Another crucial point that became very clear during the entire process of the implementation and dissemination of the neovagina creation procedure and later establishment of the UTx program was that the ability to perform such complex surgical interventions requires not only the surgical expertise to perform the relevant techniques but also the suitable ambulatory setting (a large cohort of patients with long clinical and in some cases psychological follow-up).

## Future considerations

To this date more than 60 UTx procedures, including both live donor and deceased donor procedures, have been performed worldwide. The first live birth after live donor UTx was achieved in Sweden in 2014 (Brännström et al., 2015), and three years later the first birth after deceased donor UTx followed in Brazil (Ejzenberg et al., 2019). Our collaborative experience (from 2013 to 2019) is that of 15 live donor UTx procedures in Sweden, with nine performed by classical laparotomy and six by mainly robotic-assisted laparoscopy in the donors.

In Germany, we have performed three live donor laparotomy UTx procedures. From this overall cohort of 18 procedures, 11 babies (9 in Sweden and 2 in Tübingen) have been born so far (unpublished data). To our knowledge, 6 additional births have meanwhile taken place worldwide. These include the first live birth after deceased-donor UTx (Ejzenberg et al., 2019), which occurred in Brazil in December 2017; around the same time the first live birth after live-donor UTx in the United States was reported (Testa et al., 2018). There have been also media reports of another birth in the USA, one in Serbia, one in India, and one in China.

The successful treatment of AUFI by UTx opens fascinating new horizons in modern women’s health. However, we will also encounter a number of medical and ethical challenges that have not yet been fully clarified by other transplantation procedures. Nevertheless, the advantages that arise for women with AUFI are countless. Future research activities should focus on the introduction of minimally invasive techniques in UTx that may lead to reductions in perioperative and postoperative morbidity, a shorter hospital stay, and faster return to normal daily activities and work for both the donor and the recipient. Another important area of development is the optimization of the pre-donation screening process for both live and deceased donors so as to ensure that only organs with a very good chance of graft survival and successful pregnancy will be selected for transplantation. Surveillance during pregnancy and immunosuppression, as determination of the best period for embryo transfer initiation and time to delivery are additional research aspects of major interest. Also, skipping immunosuppressive treatment may become a reality when the vision of creating a completely bioengineered uterus is translated into clinical practice (Hellström et al., 2016). To advance this goal, Mats Brännström founded the International Society for Uterus Transplantation (ISUTx; www.isutx.org) in 2017, which aims to bring together all groups of clinicians and researchers involved in establishing UTx worldwide. Currently, ISUTx focuses on organizing an annual scientific meeting and implementing a database to collect and analyse relevant data on all aspects of UTx, from selection criteria to long-term follow-up of donors, recipients, and their children.
